# Digitally assisted diagnostics of autism spectrum disorder

**DOI:** 10.3389/fpsyt.2023.1066284

**Published:** 2023-02-01

**Authors:** Jana Christina Koehler, Christine M. Falter-Wagner

**Affiliations:** Department of Psychiatry and Psychotherapy, Medical Faculty, LMU Munich, Munich, Germany

**Keywords:** autism, digital phenotyping, diagnostics, early recognition, markers

## Abstract

Digital technologies have the potential to support psychiatric diagnostics and, in particular, differential diagnostics of autism spectrum disorder in the near future, making clinical decisions more objective, reliable and evidence-based while reducing clinical resources. Multimodal automatized measurement of symptoms at cognitive, behavioral, and neuronal levels combined with artificial intelligence applications offer promising strides toward personalized prognostics and treatment strategies. In addition, these new technologies could enable systematic and continuous assessment of longitudinal symptom development, beyond the usual scope of clinical practice. Early recognition of exacerbation and simplified, as well as detailed, progression control would become possible. Ultimately, digitally assisted diagnostics will advance early recognition. Nonetheless, digital technologies cannot and should not substitute clinical decision making that takes the comprehensive complexity of individual longitudinal and cross-section presentation of autism spectrum disorder into account. Yet, they might aid the clinician by objectifying decision processes and provide a welcome relief to resources in the clinical setting.

## 1. Introduction

Autism spectrum disorder is characterized by perceptual and behavioral alterations in the areas of social interaction, communication, and restrictive, repetitive interests and behaviors (1). Symptoms in the behavioral domain continue to be mostly measured by introspective procedures in the form of self-report questionnaires, or relatively standardized behavioral observation and external reports by, e.g., a caregiver. The quality of these procedures is limited by both insufficient reliability (2) and subjectivity in both self-report and the clinical professional’s assessment during behavioral observation. The outcome quality of behavioral observations is dependent on the clinical expertise of the examiner, as well as their attention and memory at the time of documentation. Unconscious bias based on, e.g., gender (3) or socioeconomic status (4), cannot be entirely excluded. Furthermore, existing neuropsychological assessments for autism spectrum disorder are limited by insufficient or unclear specificity. In addition, the marked phenomenological heterogeneity of autism spectrum disorder presents a particular diagnostic challenge. There is little doubt, however, that early correct diagnostic classification is likely to significantly contribute to a favorable developmental trajectory (5).

Internationally, the autism diagnostic observation schedule (ADOS; 6, 7) is considered the diagnostic gold standard for autism spectrum disorder with child and adolescent populations. The instrument encompasses five modules, which are adapted to language ability and age, including a toddler module that can be applied as early as 12–30 months of age (7). Extensive certified training and regular attendance at coding seminars is required for quality assurance. The ADI-R (8), a complementary caregiver interview to assess the developmental trajectory of the child or adolescent in question, also requires extensive certified training but is not regularly used in practice due to the extensive application time (about 3 h). In sum, diagnostics is currently based on a lengthy, resource-heavy clinical process, which includes a detailed neuropsychological assessment, as well as anamnestic surveys by a multidisciplinary team to ensure the quality of diagnostic reliability through diagnostic consensus (2).

Another difficulty within the diagnostic process is the lack of phenomenological or biological diagnostic markers. Autism spectrum disorder is characterized by extremely complex and heterogeneous symptomatology. As a result, conventional statistical methods have difficulty in capturing them, making it challenging to explore improved early detection, diagnosis, and treatment options (9). In recent years, psychiatric research, and autism research in particular, have frequently turned toward more complex models that are designed to make more accurate predictions for individuals in diagnostics and treatment based on the application of machine learning. Machine learning, a subfield of artificial intelligence, can discover methods, parameters, and patterns in complex data sets, largely on its own, to train optimal predictive models, which can in turn be applied to new data sets. Such algorithms are already being successfully applied in other medical fields, e.g., early cancer detection (10).

Therefore, continued development of diagnostic standards and usability of digital applications are actively discussed (11, 12). The automated acquisition of behavioral markers is being explored in many research areas, including autism spectrum disorder, of which automated video analysis methods are particularly noteworthy. Because individuals with autism spectrum disorder represent a highly sensorily sensitive and vulnerable population, less invasive methods offer distinct advantages. In addition, the necessary scalability of a future application in diagnostics is more likely with video-based methods than, for example, with imaging methods.

Thus, digital phenotyping plays a growing role in the field of mental health, and digitally assisted diagnostics appear promising. Although the role of clinical impression formation in differential diagnosis of autism spectrum disorder remains obligatory and not set out to be replaced by digital technologies, resource reduction through technological support of clinical decision making, and hopefully a subsequent reduction in waiting times for diagnostic assessment, appears worthwhile.

Below, several innovations in digitally assisted screening and diagnostics are presented, which, within the scope of this article, are primarily based on, but not limited to, nonverbal behavioral markers. Behavioral markers possess the unique potential for a non-invasive assessment, increasing its feasibility for implementation in a challenging patient population such as autism. An ecological implementation in clinical contexts is of particular importance regarding the vast heterogeneity in autistic phenotypes and the large sample sizes needed for the exploration of stable markers across the entire spectrum. By directing our particular focus toward behavioral markers, we aim to underline the potential of these methods and argue our claim, why improvements in the digital assessment thereof are worthwhile.

There are also similar efforts in the automated detection of verbal behavioral markers, such as peculiarities in prosody or idiosyncratic utterances (13), as well as in vocalizations (14) along with a number of increasingly studied but still nonspecific biomarkers (15). Additionally, an increasing number of studies has explored the use of digital interventions in autism, allowing for the assessment *via* meta-analyses [e.g., (16–18)]. However, in the digital assessment of behavioral marker research, streamlining is lacking. In the following, we provide an overview of current digital marker research in the areas of automated capture of social interaction and synchrony, facial expressions, eye movements, and motor abnormalities.

## 2. Social interaction and synchrony

Difficulty in initiation and reciprocity in social interactions is considered one of the main features of autism spectrum disorder. Social interactions are multimodal and complex constructs that require the precise coordination and interpretation of one’s own and others’ verbal and nonverbal behaviors. Studies show that even very brief snippets of behavior by individuals with autism spectrum disorder are sufficient to leave an “odd” impression and reduce the desire for further interactions (19, 20). There is evidence that one root cause of this perceived “oddity” lies within an atypical temporal fine-tuning of verbal and nonverbal communication signals (21). Therefore, the phenomenon of social synchrony is becoming increasingly relevant in autism research and is already the focus in a wide variety of research disciplines on interpersonal processes, such as empathy or relationship formation (22). Synchrony refers to the matching of one’s own, as well as the reciprocal behaviors of others, that are coordinated either simultaneously or in specific temporal sequence patterns (e.g., antiphasic step sequence in walking).

In autism spectrum disorder, studies provide evidence of reduced synchrony across multiple modalities [e.g., movements, heartbeat, speech (22)], both between two interaction partners (INTERpersonal) and within a communicating person (INTRApersonal) (23). For example, in standardized laboratory experiments, reduced synchrony was found between children with autism spectrum disorder and their parents during joint chair rocking or pendulum movements (24, 25). However, because social interaction difficulties in autism spectrum disorder are most apparent in everyday situations, it may be useful to additionally investigate interaction patterns within more ecologically valid, naturalistic social interactions beyond the laboratory setting. Here, digital methods offer opportunities to quantify subjective impressions for the first time. To this end, we examined conversations between adults with autism spectrum disorder and control subjects using automated video analysis (26). Dyads were either mixed (one person with autism spectrum disorder and one control person) or homogeneous (two persons with autism spectrum disorder or two control persons). Again, reduced movement synchrony was found in the conversational dyads including at least one person with autism spectrum disorder; this was the case across groups and across different conversational content. Subsequent data analysis using machine learning revealed high classification accuracy based on intrapersonal movement synchrony among subjects with autism spectrum disorder in these conversational settings (12).

To investigate the potential of automated video analysis in a clinical context, we conducted a scientific study of actual diagnostic interviews within representative patient populations of two specialized outpatient clinics for adults with autism spectrum disorder (27). In each case, pairs of diagnostician and patient were examined with respect to movement synchrony. For all participating individuals, a diagnosis of autism spectrum disorder was subsequently confirmed or ruled out. Comparison of these groups revealed significant differences in synchrony during the diagnostic interviews. The reduction in synchrony was specific for the group of individuals for whom the diagnosis of autism spectrum disorder was confirmed, demonstrating that the potential for differential diagnostic decision-making within a real-world context was higher than with common questionnaire measures (27).

Initial findings of reduced synchrony in conversational dyads with children with autism spectrum disorder also suggest relevance across the lifespan and associations with symptom severity (28).

## 3. Facial expression

Facial expression characteristics in autism spectrum disorder, such as reduced mimicry or reduced frequency of certain emotional expressions (29), are already evident in childhood. Recognition of emotional facial expressions also appears to be reduced; however, the literature presents a largely inconsistent pattern. For example, age and cognitive functioning abilities seem to have an impact on the spontaneous production of emotional facial expressions, while the results significantly depend on the type of measurement (29). Similar varying effects are found for emotion recognition (30). Stereotypical facial expressions, for example, seem to be recognized considerably better than subtle expressions (31). Moreover, this reflects the heterogeneous phenotype of autism spectrum disorder, making digitally assisted analysis approaches promising for examining existing alterations in recognition and production of facial expressions in a more fine-grained manner.

Non-invasive digital data collection technologies have the advantage that they can be used as early as infancy and, unlike human raters, can detect subtle differences in facial expressions. In a recent study (32), 16–31-month-old children with and without autism spectrum disorder were shown short video clips on tablets while the integrated camera of the device recorded facial expressions and head movements of the children. Objective computer vision analyses of the recordings showed that children with autism spectrum disorder more often displayed neutral and less expressive facial expressions during attentive video viewing. Leo et al. (33) compared a self-developed algorithm for emotion recognition based on computer vision in 6–13-year-old children with autism spectrum disorder with manual assessments by various psychologists. While emotional facial expressions could be reliably detected in both cases, the algorithm proved superior in detecting more subtle differences. This is particularly relevant with regard to *camouflaging* (34), a suspected social coping strategy through the acquisition of socially adapted behaviors, which has been frequently discussed in the recent past. Despite social adaptation strategies, behavior usually remains inflexible, but conspicuities may be quite subtle and only detectable dependent on expertise. Thus, digitally assisted diagnostics could be useful especially in adolescence and adulthood, as well as in the high-functioning range of autism spectrum disorder.

A translational application for diagnostics in adulthood can be found in a study by Drimalla et al. (35). In the so-called “Social Interaction Task,” subjects interact with a virtual person, whereby the patterns in the subjects’ facial expressions and voice were analyzed for their usefulness in predicting autism spectrum disorder. Subjects with autism spectrum disorder were identified with 73% accuracy.

A feasibility study recently investigated the acceptability and practicability of smartphone-based technologies to capture and record autism-related emotional expressions and behaviors while watching videos (36). Several thousand videos and questionnaires were collected, and significant differences in attention and emotional expressions were shown to be related to risk status for autism spectrum disorder, as well as to gender and age (36). Most importantly, the study demonstrated high acceptability and feasibility, with 87.6% of the data collected through the application being usable (36).

## 4. Gaze and eye movements

Abnormalities in eye movements and in the social usage of direct gaze are important symptomatic characteristics of autism spectrum disorder and are considered so-called “red flags” in early childhood (i.e., a behavior that, in combination with other relevant behaviors, should prompt further diagnostic testing). Jones and Klin (37) have shown that gaze behavior appears largely inconspicuous in the first two postnatal months within a high-risk cohort of children with at least one sibling with autism spectrum disorder, followed by a significant reduction of direct gaze between 2 and 6 months of age. Most importantly, gaze behavior could retrospectively be used to predict the diagnosis of autism spectrum disorder given at 36 months.

Pierce et al. (38) examined gaze preference of infants with autism spectrum disorder from 14 to 42  months of age. They found a significantly pronounced preference of toddlers with autism spectrum disorder for geometric figures compared to social pictures, which was not the case for toddlers without autism spectrum disorder. Gaze preference for human faces also appears to be different for children with autism spectrum disorder. For example, eye-tracking data provide evidence that children with autism spectrum disorder fixate on certain facial areas for much shorter periods of time (39). These differential patterns in face viewing could be used to distinguish between children with and without autism spectrum disorder with a high degree of accuracy in a study of 4–11-year-old children (39).

Study findings for adulthood are similar. For example, an analysis of gaze patterns while viewing Web pages showed that adults with autism spectrum disorder and without intellectual impairment could be distinguished from adults without a diagnosis with 74% accuracy (40).

## 5. Motor difficulties

Leo Kanner described motor abnormalities in children with autism spectrum disorder, though these are not regarded as an official diagnostic criterion (41). Difficulties in gross and fine motor skills have a high prevalence in autism spectrum disorder and are increasingly moving into focus. This is not least illustrated by a change in the DSM-5 (42) according to which autism spectrum disorder can now comorbidly be diagnosed with developmental coordination disorder (dyspraxia). In addition, there appear to be correlations between motor difficulties and symptom severity, as well as cognitive functioning. In a series of motor tests in children with autism spectrum disorder between 5 and 12 years of age, Kaur et al. (43) found reduced abilities in gross and fine motor skills, greater variability in movements, and reduced interpersonal synchrony compared with a neurotypical control sample. In addition, low IQ was found to be related to marked deficits in gross and fine motor skills, whereas symptom severity was rather related to general coordination difficulties. Motor difficulties also appear to persist across the lifespan in autism spectrum disorder, although studies on this are scarce. For example, in a survey of a large sample of adults with (*n* = 2,871) and without (*n* = 10,706) autism spectrum disorder, Cassidy et al. (44) found a significantly higher rate of comorbid dyspraxia in autism spectrum disorder. Interestingly, within the group of adults without an autism diagnosis, associations between behaviors that bear a resemblance to autism spectrum disorder^1^ and the frequency of a dyspraxia diagnosis were found.

Although the prevalence of motor abnormalities in autism spectrum disorder appears to be high, they frequently remain underdiagnosed or untreated (45). However, early diagnosis and subsequent intervention is essential, as evidence hints at a link to later social functioning, as well as high levels of distress due to motor difficulties in those affected (45). Digital methods could also be of assistance in this regard (55, 56).

In an early 1998 study, Teitelbaum et al. (46) found motor abnormalities in a retrospective analysis of video recordings of 4–6-month-old infants later diagnosed with autism spectrum disorder. Among other observations, the asymmetrical use of both halves of the body during crawling, walking, or sitting became clear. Nowadays, with novel motion tracking technologies, in-depth movement analyses are possible. For example, in a study by Wedyan and Al-Jumaily (47), 12–36-month-old infants were fitted with motion sensors on their wrists while playing with a ball that was also equipped with motion sensors. Motion data from the sensors were used to train a machine learning algorithm that identified infants with autism spectrum disorder with a high degree of accuracy. Furthermore, differences in hand movements, captured with 3D optical sensors, can already distinguish between older children with and without autism spectrum disorder with high classification accuracy [up to 96.7%, see 48]. Computer vision tracking methods show differences in head movements between children with and without autism spectrum disorder (49). Such discrepancies are detectable as early as 16 months of age (50). Tablet-based movement data can also prove informative. For example, in a study of 3–6-year-old children, Anzulewicz et al. (51) found that children with autism spectrum disorder exerted higher pressure and larger gestures when interacting with a tablet, whereby these data had high classification accuracy. There are also approaches to utilize motor difficulties for diagnosis in adulthood. For example, Vabalas et al. (52) trained a machine learning algorithm that could identify autism spectrum disorder in adults with 73% accuracy based on imitation of simple isolated hand movements. In this study, prediction became even more accurate when eye movements were included.

## 6. Conclusion

The methods of digitally supported diagnostics of autism spectrum disorder described in this article promise more objective, reliable, and quicker facilitation of diagnostic clarification in the future.

For early pediatric screening, referral to specialized outpatient clinics for diagnostic clarification should be considered on the basis of the following abnormalities (so-called “red flags,” here exemplary for the second year of life): Lack of responsiveness to social cues such as eye contact, name calling, and social smiling; lack of shared joy and reduced pointing gestures; poor coordination of gaze, facial expressions, gestures, and vocalization; unusual prosody; and repetitive movements of the body or objects (53).

Future support of the screening process to increase accuracy and, more specifically, reduce the burden on resources of the current medical system by digital methods would be desirable.

Although promising, research on digital phenotyping is currently still on a basic level. With a prevalence of autism spectrum disorder of around 1% (42), available sample sizes are limited, so the findings listed must be considered preliminary. With the increasing number of research studies, distinct meta-analyses will be made possible. Since individualized approaches, such as machine learning, are dependent on the availability of large data sets, it will be crucial for future research to collect and anonymize data for open-source access.

## Author contributions

JK and CF-W wrote the original manuscript. JK translated the manuscript to English. CF-W proof-read the translation. All authors contributed to the article and approved the submitted version.

## Funding

JK was funded by a PhD scholarship of Stiftung Irene. CF-W was supported by the DFG (German Research Council; grant numbers: FA 876/3-1; 876/5-1).

## Conflict of interest

The authors declare that the research was conducted in the absence of any commercial or financial relationships that could be construed as a potential conflict of interest.

## Publisher’s note

All claims expressed in this article are solely those of the authors and do not necessarily represent those of their affiliated organizations, or those of the publisher, the editors and the reviewers. Any product that may be evaluated in this article, or claim that may be made by its manufacturer, is not guaranteed or endorsed by the publisher.

## Footnotes

^1^Such behaviors in individuals without autism spectrum disorder are often called "autistic traits." Although this term suggests a connection with autism spectrum disorder, scientific evidence to this effect is lacking.
